# Multimodality Assessment for Durable Mechanical Circulatory Support Implantation

**DOI:** 10.3390/diagnostics15222886

**Published:** 2025-11-14

**Authors:** Luca Martini, Antonio Pagliaro, Francesca Maria Righini, Massimo Mapelli, Cristina Madaudo, Nicolò Ghionzoli, Carlotta Sciaccaluga, Sonia Bernazzali, Massimo Maccherini, Serafina Valente, Giulia Elena Mandoli, Antonio Luca Maria Parlati, Matteo Cameli

**Affiliations:** 1 Department of Medical Biotechnologies, Division of Cardiology, University of Siena, 53100 Siena, Italy; luca.martini@student.unisi.it (L.M.); serafina.valente@unisi.it (S.V.); giulia.mandoli@unisi.it (G.E.M.); matteo.cameli@unisi.it (M.C.); 2 Department of Heart-Chest-Vessels, Clinical-surgical Cardiology, AOU Senese, 53100 Siena, Italy; antonio.pagliaro@gmail.it (A.P.); righini.francescamaria@gmail.com (F.M.R.); 3 Department of Critical and Rehabilitative Cardiology, Heart Failure and Clinical Cardiology, Centro Cardiologico Monzino, 20138 Milan, Italy; massimo.mapelli@ccfm.it (M.M.); almparlati@gmail.com (A.L.M.P.); 4 Department of Health Promotion, Mother and Child Care, Internal Medicine and Medical Specialities (ProMISE), University of Palermo, 90129 Palermo, Italy; 5 Department of Heart-Chest-Vessels, Surgical Therapy of the Heart Failure and of the Heart Transplantation, AOU Senese, 53100 Siena, Italy; nicologhionzoli@gmail.it (N.G.); carlotta.sciaccaluga@gmail.it (C.S.); sbernazzali@gmail.it (S.B.); maccherinim@gmail.com (M.M.)

**Keywords:** advanced heart failure, LVAD, DMCS, speckle-tracking echocardiography, right heart catheterization, cardiac magnetic resonance

## Abstract

The prevalence of advanced heart failure (AdHF) is increasing globally, driven by population aging and improved survival rates in chronic heart failure (CHF). Durable Mechanical Circulatory Support (DMCS), particularly Left Ventricular Assist Devices (LVADs), has become a cornerstone in AdHF management. However, its successful implantation requires a comprehensive preoperative evaluation integrating cardiac, hemodynamic, and systemic assessments. Echocardiography and cardiac magnetic resonance (CMR) provide critical data for risk stratification—e.g., LV ejection fraction < 25%, LV end-diastolic diameter < 60 mm, or free wall RV longitudinal strain (fwRVLS) > −14% predict poorer outcomes. Right heart catheterization (RHC) identifies hemodynamic contraindications (PVR > 6 WU, PAPi < 1.5, cardiac index < 2 L/min/m^2^), while cardiopulmonary exercise testing (CPET) remains pivotal for assessing functional reserve (peak VO_2_ < 12 mL/kg/min or <50% predicted). Systemic assessment must address renal, hepatic, oncologic, and psychiatric comorbidities that influence surgical risk. Integrating these multimodal data within a multidisciplinary framework—spanning cardiologists, cardiac surgeons, anesthesiologists, and psychologists—optimizes selection and outcomes for DMCS candidates.

## 1. Introduction

Advanced heart failure (AdHF) is defined by the European Society of Cardiology (ESC) as a condition characterized by the persistence of heart failure (HF) signs and/or symptoms, despite the use of optimal medical therapy (OMT) [1]. The prevalence of AdHF is rising, driven by an aging population with chronic heart failure (CHF) and the advanced of therapeutic strategies that improve survival. Heart transplantation (HTx) and Durable Mechanical Circulatory Support (DMCS) implantation are the only definitive treatment options available for these patients [1].

Despite clear guideline-based indications, pre-implant evaluation remains heterogeneous across centers. A standardized, multimodal framework is required to integrate imaging, hemodynamic, and systemic findings for individualized risk prediction and timing of implantation.

This review summarizes not only the diagnostic techniques but also their prognostic implications and how they guide multidisciplinary decision-making for DMCS implantation.

## 2. Durable Mechanical Circulatory Support

DMCS was initially an alternative to HTx; however, earlier models were associated with higher mortality rates. Recent advancements, particularly with the Heart Mate 3 (HM3) device, have significantly improved outcomes, showing one- and two-year survival rates of 86.6% and 82.3%, respectively. The HM3, which operates using centrifugal flow technology, is currently the only LVAD approved for implantation [2,3]. DMCS can be employed in various scenarios, including as a bridge to transplantation (BTT), bridge to recovery (BTR), bridge to decision (BTD), bridge to candidacy (BTC) or as destination therapy (DT) [4]. The device consists of the following components:

1. Inflow cannula: Connects the apex of left ventricle (LV) to the pump.
2. Outflow graft: Directs the blood to the ascending aorta.
3. Pump itself and its parameters as pump speed (PS), pump power (PP), and pulsatility index (PI): PS represents the pump’s revolutions per minutes, adjustable in order to reduce or increase the device flow (pump flow, PF). PP is the energy applied by the motor in order to achieve a certain value of PS and PF. PI represents the flow fluctuations through the time.
4. External controller: Allows real-time monitoring of DMCS functionality through key parameters.
5. Driveline (DL): Links the controller to the batteries.

**Figure 1:**
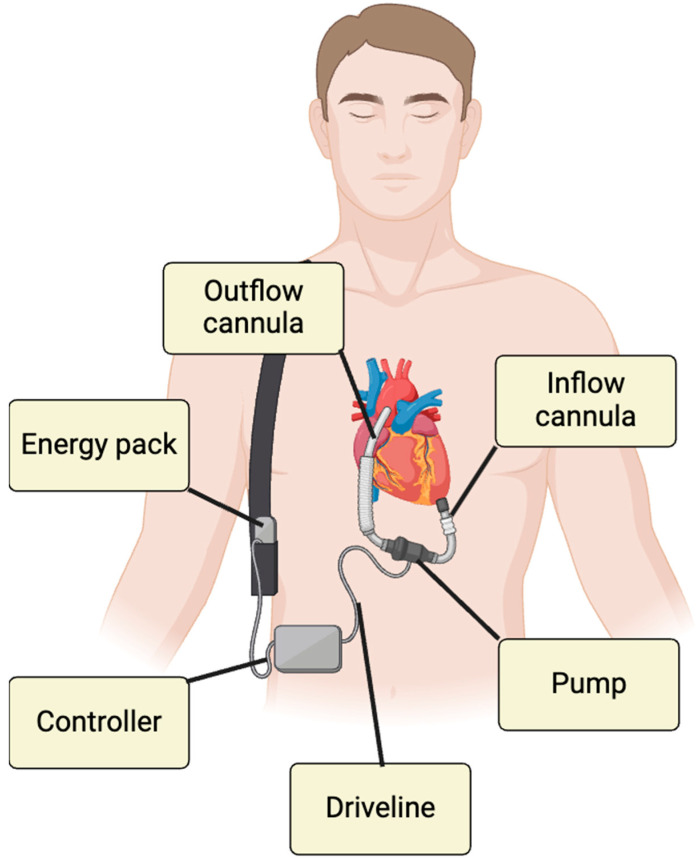
DMCS components.

These components are illustrated in Figure 1.

Beyond technological progress, optimal outcomes depend on careful patient selection. Parameters such as age, frailty, renal and hepatic function, and right ventricular performance influence whether the device is implanted as bridge to transplantation, destination therapy, or bridge to decision.

Common postoperative complications—including right heart failure, infection, pump thrombosis, and bleeding—remain major determinants of survival, underscoring the importance of accurate preoperative assessment.

## 3. Cardiac Assessment

Prior to DMCS implantation, it is essential to assess both the eligibility criteria and potential contraindications, as outlined in Table 1 [1,5].

To minimize the risk of adverse outcomes, these factors should be carefully checked, along with other clinical features associated with poor prognosis. Regarding cardiac evaluation, transthoracic and transoesophageal echocardiography (TTE and TOE, respectively) are typically the first approaches to AdHF, as they allow for comprehensive evaluation of both left and right ventricular function [6]. Firstly, left ventricle (LV) dimensions and functionality must be assessed to determine the suitability of DMCS implantation [6]. As mentioned in Table 1, one of the surgical criteria for LVAD implantation is a LV ejection fraction (EF) < 25%, which can be evaluated both in 2D and, if available, in 3D TTE, with the latter being considered more reliable in alignment with cardiac magnetic resonance (CMR) values with less test–retest variability [7,8,9]. Additionally, LV size should be evaluated in terms of both diameters—end-diastolic diameter (EDD) and end-systolic diameter (ESD)—and volumes—end-diastolic volume (EDV) and end-systolic volume (ESV). A LV end-diastolic diameter (LVEDD) < 60 mm in men or <58 mm in women predicts early post-implant mortality, while a LV ejection fraction (LVEF) < 25% remains a standard criterion for candidacy [6,10].

Where feasible, 3D TTE is again the preferred modality due to its improved accuracy and reproducibility [6,7,8,9].

Right ventricle (RV) plays a key role in the DMCS checklist, with RV dysfunction (RVD) being one of the most common post-implantation complications [11]. Due to this, patients with severe RVD are not suitable candidates for surgery [5]. RV function can be assessed using different parameters, including the tricuspid annular plane systolic excursion (TAPSE) and tissue Doppler imaging (TDI)-derived S’ [6]. However, these measures reflect only the RV longitudinal function, with classical 2D-TTE having only the right ventricular fractional area change (RVFAC) as a global systolic parameter [6,7]. RVFAC is more accurate than TAPSE in RV assessment and its reduction is an independent predictor of mortality, HF, and stroke [12]. Nevertheless, RVFAC requires a good endocardial border definition, which can be challenging to obtain in many AdHF patients [13]. To address these limitations, additional techniques have been explored, with the already mentioned 3D-TTE being the only echocardiographic technique that can evaluate RV EF [6]. In recent years speckle-tracking echocardiography (STE) has also been applied to the RV, providing two key parameters to assess RV systolic function: the RV global longitudinal strain (RV GLS) and the free wall RV longitudinal strain (fwRVLS) [6]. RV GLS includes both RV free wall and the interventricular septum (IVS), making it less specific than fwRVLS, as the IVS is strongly influenced by LV kinetics [6,14]. Several studies showed a significant correlation between fwRVLS and RV myocardial fibrosis [14]. FwRVLS values > −14% are linked with increased RVF post-DMCS implantation, while values < −11% are strongly linked with depressed RVSWI [15,16,17].

Aortic regurgitation (AR) must be assessed carefully prior to the surgery, due to its tendency to worsen postoperatively. In cases of moderate or severe AR, surgical correction or valve replacement is required [18,19]. Moderate or severe mitral stenosis should also be excluded, as it may impair DMCS filling and contribute to pulmonary hypertension (PH) [19]. Additionally, the presence of moderate or severe tricuspid regurgitation, mechanical prosthetic valves, and/or intracavitary thrombi should be documented preoperatively, as these findings are associated with poorer outcomes following implantation [19,20]. In this context, transoesophageal echocardiography (TOE) plays a key role in preoperative assessment, particularly for evaluating the left atrial appendage (LAA) and identifying thrombotic material. TOE also provides detailed visualization of valvular pathology. Furthermore, the use of contrast-enhanced (saline bubble) TOE allows for the detection of intracardiac shunts, such as atrial or ventricular septal defects that may adversely affect surgical outcomes. Any interatrial shunt identified intraoperatively must be surgically closed prior to the activation of the DMCS pump [6].

Cardiac magnetic resonance (CMR) is considered the gold standard for both LV and RV evaluation in terms of volumes and EF, offering superior accuracy in analyzing abnormal anatomies [6,21]. In addition, CMR can identify fibrotic areas with both Late Gadolinium Enhancement (LGE) and mapping sequences, proving crucial insights into cardiac structure and function, patients’ prognosis, and their risk of sudden cardiac death [22,23,24,25]. Furthermore T1 mapping can be altered in hypertrophic and dilated cardiomyopathy (HCM and DCM, respectively) even before LGE, demonstrating its superior sensitivity [26,27]. This increased sensitivity has also been demonstrated in cases of myocardial iron overload, where both T1 mapping and T2* relaxometry play a complementary role in tissue characterization [28]. Extra-cellular volume (ECV) is another novel parameter, which is used to assess extracellular fibrosis, being related with poor prognosis in cases of HF and cardiac amyloidosis [29,30].

Computed Tomography (CT) is another second level imaging modality that is particularly useful for the detailed evaluation of mediastinal anatomy and the spatial relationships between thoracic organs, owing to its high spatial sensitivity [31]. These features can be useful both to rule out ventricular thrombotic appositions or congenital cardiopathies, and to evaluate aortic root and main vessels [32,33]. Novel software also allows a non-invasive examination of coronary stenosis and fractional flow reserve [1,6].

The therapeutic workup for AdHF includes a comprehensive assessment of the pulmonary circulatory system. Severe and irreversible PH is a contraindication to both HTx and DMCS implantation [1]. This can be evaluated with right heart catheterization (RHC) using the Swan–Ganz catheter (SGC) [34]. SGC can measure systolic, mean, and diastolic pulmonary pressure (sPAP, mPAP, and dPAP) as well as pulmonary capillary wedge pressure (PCWP) [35]. RHC allows the measurement of hemodynamic parameters that reflect myocardial function. Cardiac output (CO), stroke volume (SV), and cardiac index (CI) can be quantified using both the thermodiluition technique and the Fick principle [34,35]. RHC also allows for the calculation of derived parameters, such as pulmonary vascular resistances (PVR) and the transpulmonary gradient (TPG), that help the identification of patients at high surgical risk. In addition, it provides important parameters for RV assessment, including the right ventricular stroke work index (RVSWI), a surrogate for RV systolic function, and the pulmonary artery pulsatility index (PAPi), which is strongly associated with postoperative survival [34,35,36,37,38,39]. Hemodynamic thresholds strongly influence eligibility. A cardiac index (CI) < 2 L/min/m^2^, mean pulmonary pressure (mPAP) > 25 mmHg, pulmonary vascular resistance (PVR) > 6 Wood units, or transpulmonary gradient (TPG) > 15 mmHg indicates advanced and possibly irreversible pulmonary hypertension [34,35]. Derived indices such as the pulmonary artery pulsatility index (PAPi)—with values < 1.5 predicting early right ventricular failure—and the right ventricular stroke work index (RVSWI) < 400 mmHg·mL/m^2^ further refine prognostic assessment [34,35,36,37,38,39].

The cardiopulmonary exercise test (CPET) is a comprehensive, multiparametric tool that evaluates cardiac, respiratory, and metabolic functionality. A severe reduction in peak oxygen consumption (pVO_2_) is not only a diagnostic criterion for advanced heart failure (AdHF) but also a key factor in determining patients’ eligibility for DMCS implantation [1].

CPET remains one of the strongest prognostic tools for both heart transplantation and LVAD implantation. A peak VO_2_ < 12 mL/kg/min (or <50% of predicted) and a VE/VCO_2_ slope > 35 are independent predictors of mortality [40,41]. A hypotensive response during exercise (systolic blood pressure drop >10 mmHg) identifies high, 90-day mortality after LVAD implantation [40,41]. Combining CPET and RHC data refines selection by linking functional limitation to objective hemodynamic burden.

## 4. Systemic Assessment

The assessment of kidney function is recommended by 2023 International Society of Heart and Lung Transplantation (ISHLT) guidelines [19]. Renal glomerular function is usually evidenced by serum creatinine and the glomerular filtration rate (GFR), which can be calculated using different formulae according to creatinine levels with CKD EPI being the most commonly used due its less biased GFR estimation [42,43]. Serum creatinine is commonly used to assess worsening renal functionality (WRF) but is limited by its dependence on patient’s age, diet, gender, and muscle mass [44]. Blood urea nitrogen (BUN), which reflects serum urea concentration, has emerged as a strong prognostic marker in HF, showing higher predictive values for morbidity and mortality compared to serum creatinine [45,46]. Cystatin C is another biomarker that correlates well with GFR, in diabetic and ischemic patients in particular, being independent of age, body mass, and nutritional status [47,48,49]. Albuminuria has been associated with an increased risk of mortality and HF-related hospitalization, independent of creatinine levels and GFR, and is considered an early biomarker of renal impairment [50]. According to ISHLT guidelines, severe renal impairment (eGFR < 30 mL/min/1.73 m^2^), hepatic cirrhosis, or active malignancy with life expectancy < 2 years represents absolute or relative contraindications to DMCS [19]. In terms of tubular function, *N*-Acetyl Beta Glucosaminidase (NAG) is one of the earliest biomarkers studied. Elevated NAG levels have been independently associated with increased mortality and HF hospitalizations, regardless of GFR [51]. Renal ultrasonography (RUS) provides morphological information such as kidney dimensions and cortical thickness, parameters that are typically reduced in chronic kidney disease (CKD) [52]. Moreover, color Doppler imaging enables the assessment of the renal resistive index (RRI), a hemodynamic parameter that rises in the presence of high-grade fibrosis, making it a risk factor for the progression of kidney disease [53]. Notably, even the evaluation of pulsatility in renal veins has shown a correlation with death and HF hospitalization [54]. Finally, both nuclear imaging and magnetic resonance (MR), can be employed to assess GFR. MR with blood oxygenation level-dependent (BOLD) sequences enable the evaluation of renal parenchymal oxygenation, offering additional insights into renal functional status [55,56].

Evaluation of nutritional and frailty indices—such as unintentional weight loss, handgrip strength, and gait speed—should complement the systemic assessment, as they correlate with postoperative recovery and survival [19].

The presence of atherosclerotic plaques must be evaluated in peripheral, carotid, and vertebral systems in order to prevent post-extracorporeal circulation stroke [19]. Ultrasonography (US) is the first recommended approach to evaluate plaques and their distribution with good specificity [57]. MR is the gold standard for the evaluation of plaque characterization, as it allows for the identification of neovascularization and differentiation in plaque components, including the fibrosus cap [57]. CT, on the other hand, offers the highest spatial resolution, capable of identifying 1 mm lesions, but requires intravenous contrast administration that can potentially impair renal functions [57].

According to ISHLT guidelines, coagulation status should also be assessed preoperatively, measuring PT/INR, aPTT, platelet count and screening for thrombophilia-related conditions (e.g., Factor V Leiden, anti-phospholipid antibodies, etc.) [19].

All patients should be screened for diabetes mellitus (usually via HbA1c measuring) and in the case of positivity, a comprehensive evaluation of diabetes-related end-organ damage is necessary, including an assessment for retinopathy, neuropathy, nephropathy, and vasculopathy [19]. Peripheral neuropathy is usually confirmed by electrodiagnostic studies, despite not impacting on treatment decisions [58]. Regarding retinopathy, the ophthalmologic evaluation includes the assessment of visual acuity and intraocular pressure, and dilated funduscopic examination [59].

Malignancies may represent a contraindication for DMCS if the estimated life expectancy is less than two years. Therefore, it is necessary to evaluate the presence of malignant lesions using both imaging and biochemical markers [19]. Regarding gastrointestinal neoplasia, in cases of a recent history of gastrointestinal bleeding, melena, unexplained iron deficiency anemia, or premalignant polyps, the screening is usually performed via upper and lower endoscopy [19].

Similarly to the assessment of candidates for HTx a comprehensive psychological analysis should be performed. Furthermore, if drug dependence is suspected, a toxicological analysis should be carried out [19].

The multidisciplinary heart team plays a pivotal role in optimizing DMCS outcomes. The cardiologist and imaging specialist provide functional and structural assessments, and the cardiac surgeon evaluates technical feasibility and concomitant valve disease, while the anesthesiologist and intensivist manage perioperative optimization [19]. Psychologists and social workers assess adherence, cognitive status, and family support. Regular multidisciplinary meetings have been associated with lower rates of postoperative right heart failure and rehospitalization, confirming that team-based evaluations are as crucial as device selection itself [1,19].

## 5. Complications Related to DMCS Implantation

Cardiac tamponade is a clinically significant postoperative complication. It can occur early, within 24 h, typically due to postoperative hemorrhage, or late, 5–7 days after cardiac surgery, as a result of multifactorial processes [60]. Conventional echocardiographic markers, such as pronounced respiratory variation in mitral inflow, are less reliable in patients receiving positive-pressure ventilation. Furthermore, continuous left ventricular unloading by an LVAD can further obscure classic signs [61]. Consequently, establishing the diagnosis relies on vigilant clinical assessments combined with serial hemodynamic and echocardiographic evaluations.

RV dysfunction is among the most common and prognostically adverse complications after LVAD implantation, representing a leading cause of early mortality. RV failure may be transient, related to cardiopulmonary bypass, RV ischemia, or tachyarrhythmia, or it may be precipitated by excessive LVAD speeds. Increased LV output can induce RV failure through two primary mechanisms: (1) volume overload of the RV due to increased systemic flow; (2) impaired RV contractility caused by interventricular septal (IVS) displacement toward the LV resulting from excessive LVAD drainage [62,63]. Echocardiography remains the cornerstone of RV assessment in LVAD candidates. Multiple parameters have been associated with postoperative RV failure, including TAPSE, FAC, RV/LV size ratios, tricuspid annular dilation, TR severity, tissue Doppler S′/E′ velocities, RV myocardial performance index, and RV longitudinal strain, even though the predictive accuracy of individual indices remains limited [64,65]. Hemodynamic metrics, such as the pulmonary artery pulsatility index (PAPi) and right ventricular stroke work index (RVSWI), are useful but constrained by preload/afterload dependence and inter-cohort variability [64,66]. Observational studies indicate that combining free wall RV longitudinal strain, PAPi, and biomarkers such as NT-proBNP improves preoperative discrimination for post-implant RV failure, although no single metric is sufficient to guide management in isolation. Accordingly, current research emphasizes multimodal risk stratification, integrating imaging-based strain, invasive hemodynamics, and circulating biomarkers. Preventive strategies under investigation include perioperative LVAD speed optimization using combined echo-hemodynamic ramp testing (pump speed is incrementally adjusted while monitoring ventricular dimensions and hemodynamics), the selective use of temporary RV mechanical support in high-risk patients, and tailored pharmacologic unloading to mitigate early RV dysfunction [67]. Late RV failure may result from intrinsic RV cardiomyopathy or maladaptive responses to excessive LVAD output. In addition to RV dysfunction, aortic valve-related complications, particularly AR, can further compromise LVAD function.

AR, de novo or as a paravalvular leak after valve replacement, is increasingly recognized as a relevant issue. Standard Doppler indices are often unreliable in this population, as continuous-flow physiology may cause regurgitation to persist throughout the cardiac cycle. Commonly proposed markers (short pressure half-time, dense continuous-wave Doppler signal, large vena contracta, diastolic flow reversal in the descending aorta, or high regurgitant fraction) may underestimate severity in LVAD patients [18,68]. Ramp testing and emerging LVAD-specific Doppler surrogates, such as outflow cannula systolic/diastolic velocity ratios or diastolic acceleration, provide useful dynamic and quantitative information to corroborate significant AR [18,69]. The reported incidence of clinically relevant AR after LVAD ranges from approximately 25–40% within the first postoperative year. Optimal management, surgical repair, or closure at the implant versus delayed percutaneous or transcatheter solutions (including TAVR in selected cases) remains debated, with evidence currently limited to retrospective series and cohort analyses [68,70].

## 6. Conclusions

**Figure 2:**
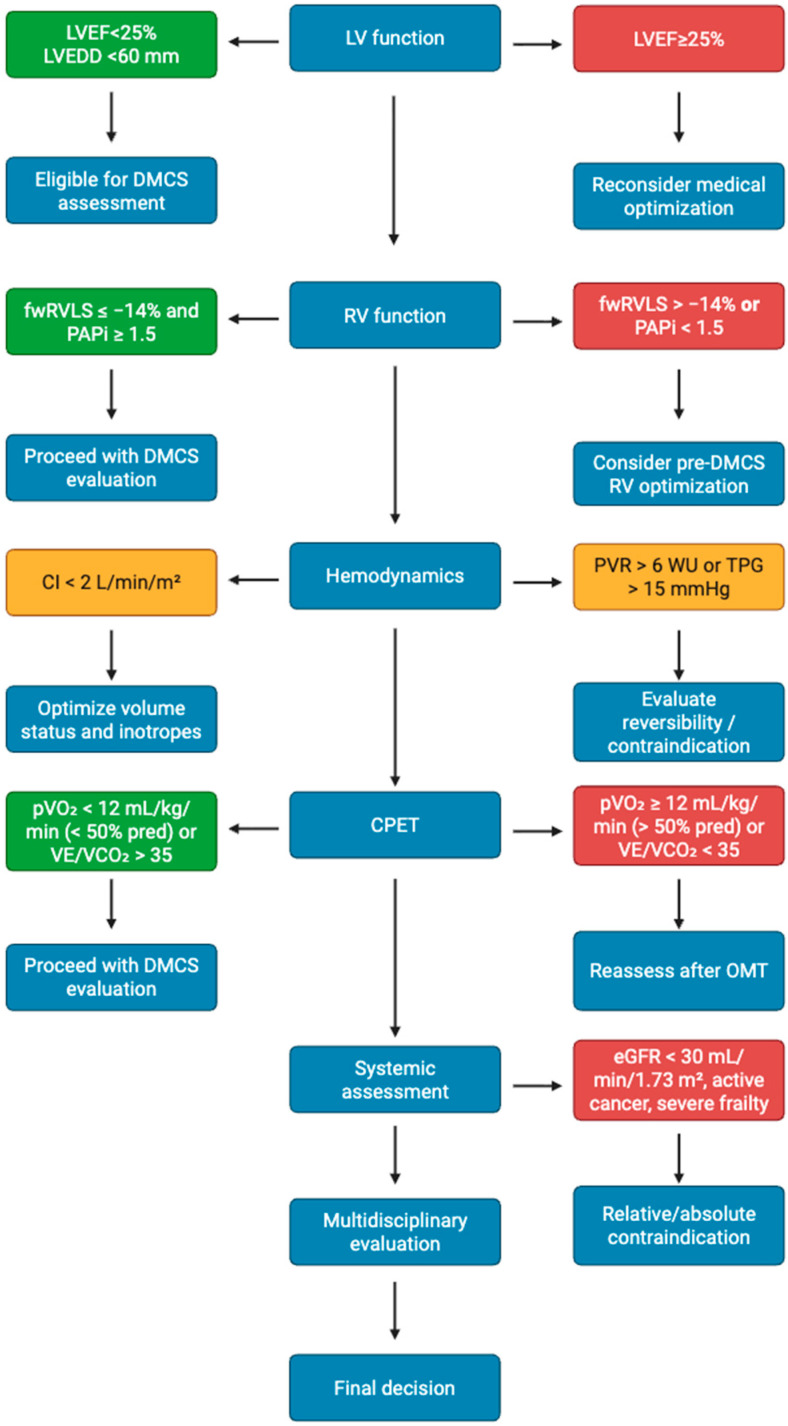
Proposed multimodality checklist for DMCS candidacy.

Preoperative evaluation for DMCS implantation must integrate multimodality imaging, hemodynamic studies, and systemic assessments into a unified multidisciplinary process (Figure 2). This structured approach improves patient selection and reduces complications, thereby aligning with the primary therapeutic goal: to restore function and quality of life. Future research should focus on integrating imaging biomarkers, hemodynamic indices, and artificial intelligence-based predictive models to personalize DMCS candidacy and timing.

## Figures and Tables

**Table 1:** DMCS implantation indications and contraindications.

Indications	Contraindications
LVEF < 25% and unable to exercise for HF or, if able to perform cardio-pulmonary exercise testing, doing so with peak VO_2_ < 12 mL/kg/min and/or <50% predicted value	Severe right ventricle dysfunction
≥3 HF hospitalizations in previous 12 months without an obvious precipitating cause	Pulmonary vascular resistances > 6 WU
Dependence on IV inotropic therapy or temporary MCS	Contraindications to long term anticoagulation
Progressive end-organ dysfunction (worsening renal and/or hepatic function, type II pulmonary hypertension, or cardiac cachexia) due to reduced perfusion and not to inadequately low ventricular filling pressure (PCWP ≥ 20 mmHg and SBP ≤ 90 mmHg or cardiac index ≤ 2 L/min/m^2^	Intractable ventricular arrhythmias
	Active infection
	Severe systemic disorders
	Severe renal dysfunction
	Neuropsychiatric illness
	Severely insulin-dependent diabetes mellitus
	Liver cirrhosis
	Extreme frailty
	Poor social support
	Pregnancy

LVEF: left ventricle ejection fraction; HF: heart failure; VO_2_: oxygen consumption; IV: intravenous; MCS: mechanical circulatory support; PCWP: pulmonary capillary wedge pressure; SBP: systolic blood pressure; and WU: Woods unit.

## Data Availability

No new data were created or analyzed in this study.
